# Generation and characterization of *Ccdc28b* mutant mice links the Bardet-Biedl associated gene with mild social behavioral phenotypes

**DOI:** 10.1371/journal.pgen.1009896

**Published:** 2022-06-02

**Authors:** Matías Fabregat, Sofía Niño-Rivero, Sabrina Pose, Magdalena Cárdenas-Rodríguez, Mariana Bresque, Karina Hernández, Victoria Prieto-Echagüe, Geraldine Schlapp, Martina Crispo, Patricia Lagos, Natalia Lago, Carlos Escande, Florencia Irigoín, Jose L. Badano

**Affiliations:** 1 Human Molecular Genetics Laboratory, Institut Pasteur de Montevideo, Montevideo, Uruguay; 2 INDICyO Institutional Program, Institut Pasteur de Montevideo, Montevideo, Uruguay; 3 Departamento de Fisiología, Universidad de la República, Montevideo, Uruguay; 4 Neuroinflammation and Gene Therapy Laboratory, Institut Pasteur de Montevideo, Montevideo, Uruguay; 5 Metabolic Diseases and Aging Laboratory, Institut Pasteur de Montevideo, Montevideo, Uruguay; 6 Departamento de Histología y Embriología, Facultad de Medicina, Universidad de la República, Montevideo, Uruguay; 7 Laboratory Animal Biotechnology Unit, Institut Pasteur de Montevideo, Montevideo, Uruguay; University of Iowa, UNITED STATES

## Abstract

CCDC28B (coiled-coil domain-containing protein 28B) was identified as a modifier in the ciliopathy Bardet-Biedl syndrome (BBS). Our previous work in cells and zebrafish showed that CCDC28B plays a role regulating cilia length in a mechanism that is not completely understood. Here we report the generation of a *Ccdc28b* mutant mouse using CRISPR/Cas9 (*Ccdc28b mut*). Depletion of CCDC28B resulted in a mild phenotype. *Ccdc28b mut* animals *i)* do not present clear structural cilia affectation, although we did observe mild defects in cilia density and cilia length in some tissues, *ii)* reproduce normally, and *iii)* do not develop retinal degeneration or obesity, two hallmark features of reported BBS murine models. In contrast, *Ccdc28b mut* mice did show clear social interaction defects as well as stereotypical behaviors. This finding is indeed relevant regarding *CCDC28B* as a modifier of BBS since behavioral phenotypes have been documented in BBS. Overall, this work reports a novel mouse model that will be key to continue evaluating genetic interactions in BBS, deciphering the contribution of *CCDC28B* to modulate the presentation of BBS phenotypes. In addition, our data underscores a novel link between *CCDC28B* and behavioral defects, providing a novel opportunity to further our understanding of the genetic, cellular, and molecular basis of these complex phenotypes.

## Author summary

BBS is caused by mutations in any one of 22 genes known to date. In some families, BBS can be inherited as an oligogenic trait whereby mutations in more than one BBS gene collaborate in the presentation of the syndrome. In addition, *CCDC28B* was originally identified as a modifier of BBS, whereby a reduction in CCDC28B levels was associated with a more severe presentation of the syndrome. Different mechanisms, all relying on functional redundancy, have been proposed to explain these genetic interactions. The characterization of BBS proteins supported this functional redundancy hypothesis: BBS proteins play a role in cilia maintenance/function and subsets of BBS proteins can even interact directly in multiprotein complexes.

We have previously shown that CCDC28B also participates in cilia biology regulating the length of the organelle: knockdown of *CCDC28B* in cells results in cilia shortening and targeting *ccdc28b* in zebrafish also results in early embryonic phenotypes characteristic of other cilia mutants. In this work, we generated a *Ccdc28b* mutant mouse to determine whether abrogating *Ccdc28b* function would be sufficient to cause a ciliopathy phenotype in mammals, and to generate a tool to continue dissecting its modifying role in the context of BBS. Overall, *Ccdc28b* mutant mice presented a mild phenotype, a finding fully compatible with its role as a modifier, rather than a causal BBS gene. In addition, we found that *Ccdc28b* mutants showed behavioral phenotypes, similar to the deficits observed in rodent autism spectrum disorder (ASD) models. Thus, our results underscore a novel causal link between *CCDC28B* and behavioral phenotypes in mice.

## Introduction

Bardet-Biedl syndrome (BBS; OMIM 209900) is a rare disorder characterized by retinal degeneration, polydactyly, intellectual disability, gonadal/renal malformations and obesity among other features [1]. BBS is a genetically heterogeneous condition with 22 genes known to cause the disease to date (*BBS1*-*BBS22*; [2,3] and references within). All BBS-causing genes encode proteins important for the formation/maintenance of primary cilia [4–14], complex organelles that have been shown to function as hubs for signaling (see for example [15,16]). Therefore, BBS is a ciliopathy, a term used to group several human conditions that are caused by ciliary dysfunction and share, to different degrees, a set of characteristic phenotypes [17,18]. While in most families BBS is inherited in an autosomal recessive manner, genetic interactions between BBS genes, modulating both penetrance and expressivity, also support an oligogenic model of inheritance [19–28].

The functional characterization of BBS proteins has provided a cellular/molecular explanation to the oligogenicity observed in BBS, a phenomenon that typically relies on the presence of complementary pathways, complexes and/or some degree of functional redundancy [29]. In this context, BBS proteins present a significant functional overlap and can even interact directly forming multiprotein complexes. Eight BBS proteins form the BBSome, a complex that mediates traffic of ciliary components [6,10,13,30–33]. Another group of BBS proteins (BBS6, BBS10 and BBS12) have a chaperone activity critical for BBSome assembly [34], while others are important for BBSome recruitment to membranes [6] or to regulate the movement of the complex in and out of cilia [35]. Moreover, cilia are composed of more than 1000 proteins with at least four main functional complexes including the BBSome, the transition zone, and two intraflagellar complexes for anterograde and retrograde transport respectively. Thus, mutations in different genes and ciliary modules can contribute to cilia dysfunction and therefore to the pathogenesis of different ciliopathies (reviewed in for example [15,36]).

As mentioned, oligogenicity is typically caused by some degree of functional redundancy. Thus, the identification of proteins that could physically/functionally interact with the BBS proteins was used both to gain insight on the cellular basis of BBS and to identify novel candidates that could contribute to the pathogenesis of the syndrome. In that context, CCDC28B (coiled-coil domain-containing protein 28B), a protein of unknown function at the time, was identified as an interactor of both BBS4 and other known BBS proteins (BBS1, BBS2, BBS5, BBS6, BBS7 and BBS8) [37]. Importantly, a sequencing screen looking for *CCDC28B* mutations in a BBS patient cohort identified a synonymous single base change at the penultimate base of exon 3 (the gene is composed of 6 exons). A minigene analysis demonstrated that this change affected exon 3 definition on the 5’ end favoring the production of an aberrant mRNA lacking the first 5 bases of exon 3, which results in a frameshift and the introduction of a premature stop codon (PTC). Thus, the net result of this mutation was shown to be a decrease (but not elimination) in *CCDC28B* mRNA levels. Importantly, while this mutation was not sufficient to cause BBS, it was shown that the resulting reduction in CCDC28B levels, in a genetic background with mutations in *bona fide* BBS genes, correlated with a more severe presentation of the syndrome [37]. Overall, the data showed that this gene could play a modifier role in BBS, at least in some patient cohorts [21,37–39].

Since that original work, we have shown that CCDC28B also plays a role in cilia. Knockdown of *CCDC28B* in hTERT-RPE cells results in shortened cilia and a reduction in the percentage of ciliated cells. Targeting *ccdc28b* in zebrafish results in a distinct external phenotype characterized by a shortened body axis, increased body curvature, craniofacial and pigmentation defects and smaller eyes, phenotypes that have been described in other cilia zebrafish mutants, including BBS mutants ([40,41] and references within). Furthermore, the analysis of different ciliated tissues in zebrafish morphant embryos showed a clear reduction in both the number and length of cilia [40–42]. While the mechanism by which CCDC28B modulates cilia is still not completely understood, we have uncovered relevant protein-protein interactions. We were able to show that CCDC28B modulates cilia length, at least in part, through an interaction with SIN1, a member of mTORC2, independently of the mTOR complex [42]. More recently, we showed that the molecular motor kinesin 1 is also involved in cilia length regulation by controlling CCDC28B sub-cellular localization [41]. Altogether our data provided insight into the cellular basis of the CCDC28B modifier effect: a reduction in CCDC28B levels, as reported in patients, could affect cilia at least to some extent, thus contributing to BBS pathogenesis when present in a BBS mutant genetic background. However, the data also raised the question of whether other types of mutations (i.e. null mutations) in *CCDC28B* could be sufficient to cause a ciliopathy.

In this work we aimed to generate a *Ccdc28b* knockout mouse that would i) allow us to determine whether loss of function of this gene is sufficient to cause cilia dysfunction and associated phenotypes in mammals, and ii) serve as a tool to continue studying its modifier effect in the context of BBS. We therefore targeted *Ccdc28b* in mice using CRISPR/Cas9 and performed an in-depth phenotypic characterization of mutant animals (*Ccdc28b mut*). We focused on phenotypes that have been described for BBS mouse mutant lines, which include the development of obesity driven by hyperphagia, and retinal degeneration. We show that depletion of CCDC28B is not sufficient to cause overt ciliary defects in cells or tissues, although a trend towards presenting a reduction in the percentage of cilia and mild cilia length defects were seen in a subset of the analyzed tissues. In agreement with this observation, *Ccdc28b mut* animals are viable, reproduce at mendelian rates and do not develop obesity or show signs of photoreceptor loss. However, *Ccdc28b mut* mice show social behavior defects, phenotypes that are being documented in both BBS patients and animal models (see for example [43–49]). Our work generated a novel genetic model that recapitulates certain phenotypes observed in BBS patients, and that will allow further dissection of the genetic, cellular, and molecular basis of complex behavioral phenotypes.

## Results

### Generation of a Ccdc28b mouse model

**Fig 1:**
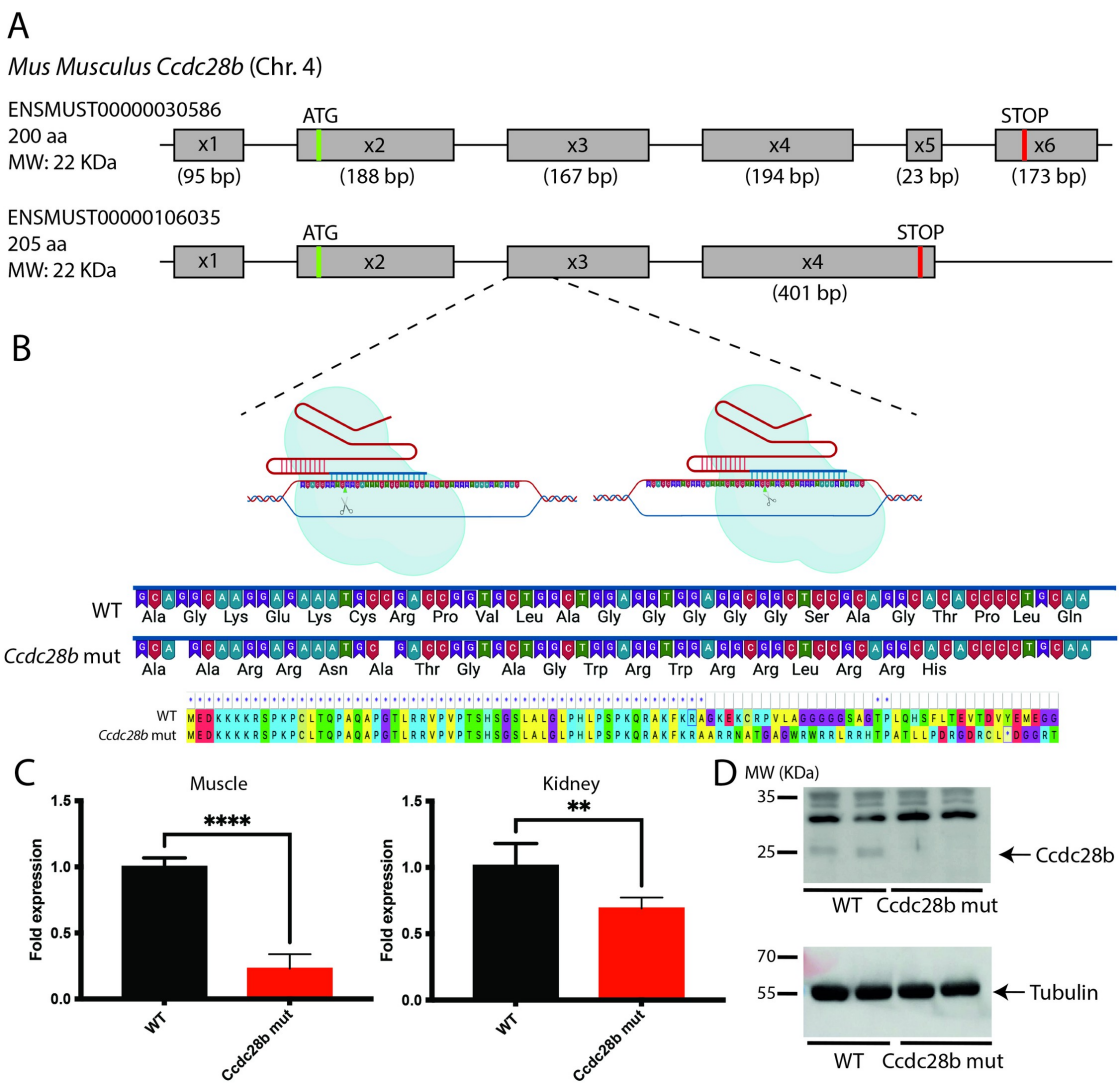
Targeting *Ccdc28b* in the mouse. **A)** Schematic representation of murine *Ccdc28b* genomic structure, showing exon distribution and the two main reported protein coding ORFs that encode two isoforms differing in their C-terminal sequences. **B)** Graphic representation showing the two gRNAs used in this study targeting the 5’ end of exon 3. The selected mutation comprises two single base deletions leading to a frameshift and the introduction of a PTC. **C)** Real-time quantitative PCR was used to show a significant reduction in *Ccdc28b* mRNA levels, likely due to NMD-mediated degradation of PTC containing mRNA. One adult male and one adult female of each genotype were used, and each sample was run in triplicates. Expression of *Gapdh* was used for normalization. Results are shown as fold change compared to *wt* control animals. ** = *P* < 0.01 and *** = *P* < 0.001. **D)** Western blot analysis of adult female muscles showing that a band corresponding to the CCDC28B expected molecular weight (aprox. 22 kDa) is depleted in *Ccdc28b mut* animals. Full gels are shown in S2 Fig.

The mouse *Ccdc28b* gene (Gene ID 66264) is located on chromosome 4 and is composed of six exons with an open reading frame spanning from exon 2 to exon 6. Although several splicing isoforms have been reported (*Ensembl* ENSMUSG00000028795), as in humans, only two transcripts are predicted to encode full length proteins in mice. These are proteins of 200 and 205 amino acids respectively, differing in their C-terminal regions (Fig 1A). To generate a knockout *Ccdc28b* murine line we choose to target exon 3, which is shared by all reported transcripts and coding isoforms, and is the exon affected by the mutation described in humans [37]. We designed two gRNAs to target the 5`end of exon 3 (Fig 1B) and verified their efficiency in targeting *Ccdc28b* by transiently transfecting a murine NIH3T3 cell line stably expressing CAS9 and performing a heteroduplex analysis. We then injected 294 zygotes with *Cas9* mRNA and our two gRNAs. Upon analysis of 12 animals by PCR amplification of exon 3 from genomic DNA and sequencing, we were able to identify several mutations in six mice (50% mutation rate). We crossed two of those founder mice with C57BL/6J females (Jackson Lab stock # 000664) to segregate the mutations, and finally, we chose to continue our work with a mutation consisting of two one base pair deletions at the 5’ end of exon 3, thus predicted to result in a frameshift and a PTC (Fig 1B). This mutated *Ccdc28b* transcript could potentially encode an 88 amino acid protein composed of the first 56 amino acids of CCDC28B followed by 32 novel residues, although mutant mRNA is expected to be targeted by nonsense-mediated decay (NMD) due to the PTC (Fig 1B). We crossed a male mouse carrying the selected mutation with C57BL/6J females to start the colony and performed two rounds of crossing before starting the characterization of the line, which from that point onwards we maintained by crossing homozygous mutant animals.

As a first step we aimed to confirm that the expression of *Ccdc28b* was abrogated. As mentioned, the mutation introduces a PTC likely targeting the mRNA for NMD. We therefore performed qRT-PCR using *Ccdc28b* specific primers and cDNA obtained from different tissues (*Ccdc28b* is widely expressed; [50]). As expected, we observed a significant reduction in *Ccdc28b* mRNA levels (Fig 1C), although at different degrees between muscle and kidney likely reflecting reported differences in NMD efficiency between tissues [51]. We also PCR-amplified *Ccdc28b* from cDNA of E14 embryos (both *Ccdc28b mut* and C57BL/6J *wt*) with primers located at the far most 5’ and 3’ ends of the reported isoforms and sequenced the PCR products using Oxford Nanopore technology taking advantage of its long-read sequencing. We detected the expected mutations and did not find any evidence of novel alternative transcripts in *Ccdc28b mut* samples (S1 Fig). Next, we evaluated CCDC28B at the protein level by western blot: the expected ~22 KDa CCDC28B band was absent in the *Ccdc28b mut* samples (Figs 1D; S2 for full gels and muscle blot). Thus, our results indicate that we were able to generate a mutant *Ccdc28b* mouse line (*Ccdc28b mut*).

### Depletion of CCDC28B does not result in overt ciliary defects but may modulate cilia length in a tissue dependent manner

**Fig 2:**
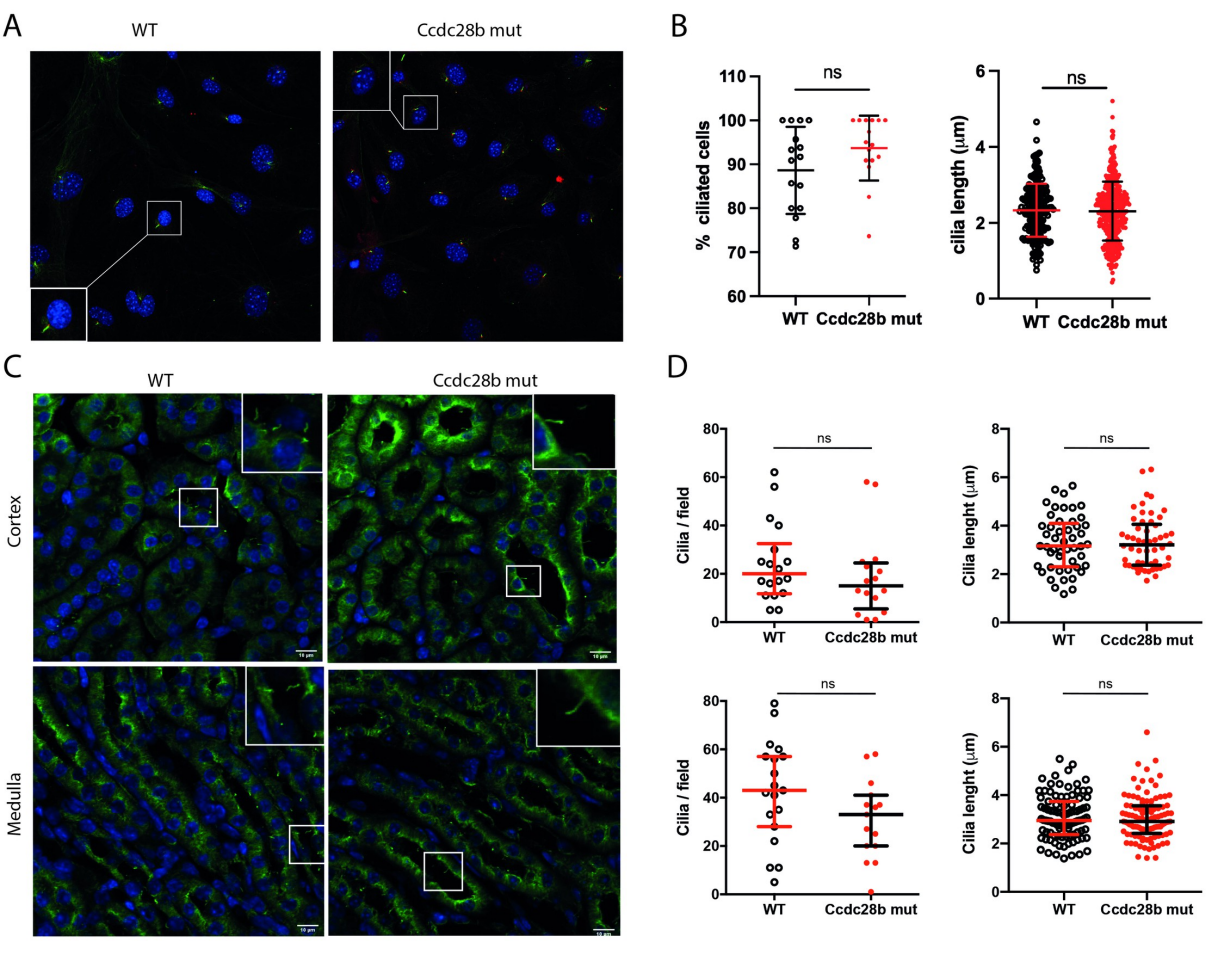
Characterization of cilia in *Ccdc28b mut* cells and tissue. **A)** Confocal images of *wt* and *Ccdc28b mut* MEFs. DAPI (blue), acetylated tubulin (green) and γ-tubulin (red) were used to visualize nuclei, ciliary axoneme and basal body, respectively. **B)** Cilia length was measured in more than 190 cilia from *wt* and *Ccdc28b mut* MEFs. Data are presented as individual values plus mean ± SD. **C)** Confocal images of *wt* and *Ccdc28b mut* kidneys (adult females were used). The upper panels correspond to kidney cortex, and the lower panels correspond to kidney medulla. DAPI (blue) and anti-acetylated tubulin (green) were used to visualize nuclei and cilia respectively. **D)** Quantification of number of cilia per field and cilia length. Data did not have normal distribution and are shown as individual values plus median with interquartile range. Despite no significant differences were found there is a trend towards a reduction in cilia density in both regions in *Ccdc28b mut* kidneys. ns: not significant.

To begin the characterization of the mutant line we first focus on the known function of CCDC28B. As previously mentioned, our work in hTERT-RPE cells and zebrafish uncovered a role for CCDC28B in cilia whereby its depletion resulted in a reduction in ciliary length as well as a reduction in the percentage of ciliated cells [40–42]. We first focused on measuring cilia length and quantifying the proportion of cilia in mouse embryonic fibroblasts (MEFs) obtained from both *Ccdc28b mut* and C57BL/6J (*wt*) E14 embryos. We quantified the proportion of ciliated cells by counting nuclei and cilia per field, using anti γ-tubulin and anti-acetylated tubulin antibodies to visualize the basal body and ciliary axoneme respectively. We measured cilia length by analyzing at least eight randomly selected fields from each of *Ccdc28b mut* and *wt* MEFs. Our data showed that depletion of CCDC28B did not result in shortened cilia nor affected ciliation in these cells: the median cilia length of *wt* MEFs was 2.33 ± 0.70 μm compared to 2.31 ± 0.78 μm in *Ccdc28b mut* cells (*P* = 0.6522; Fig 2A and 2B).

**Fig 3:**
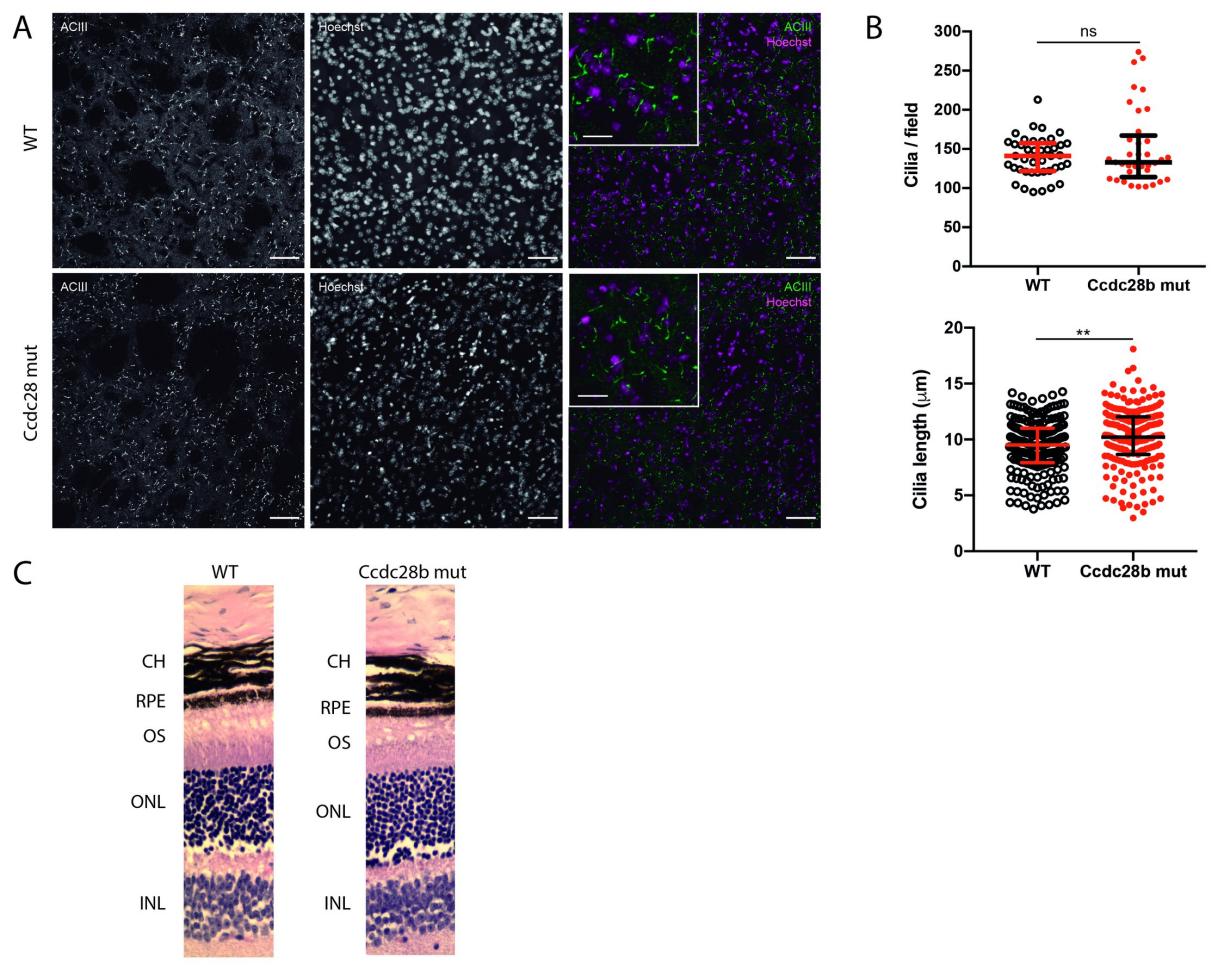
Analysis of brain cilia and the retina in *Ccdc28b mut* animals. **A)** Confocal images showing cilia in the brain striatum. DAPI (magenta) and anti-Type III adenylyl cyclase (ACIII, green) were used to visualize nuclei and ciliary axoneme respectively. Bars in main pictures and insets correspond to 50 and 20 microns respectively. Representative images are shown. **B)** Quantification of number of cilia per field (at least three pictures per animal, and three animals per genotype) and cilia length (one picture per animal, three animals per genotype) in striatum. One male and one female were used for initial assessment (not shown) and three females were then used for quantification. Results are represented as individual values plus median with interquartile range. ** = *P* < 0.005. **C)** Hematoxylin and eosin-stained retinal sections of *wt* control and *Ccdc28b mut* mice (adult females were used in the analysis). No structural differences were observed in the photoreceptor layer. INL = inner nuclear layer; ONL = outer nuclear layer; OS = Outer segment; RPE = retinal pigment epithelium; CH = choroid.

*Ccdc28b mut* mice reproduce normally although when measuring weeks at first mating *Ccdc28b mut* females showed a delay (approximately 14 weeks) compared to C57BL/6J females (approximately 7 weeks). We then evaluated cilia in the kidney, a tissue where cilia are readily observed. Kidneys from both *wt* control and *Ccdc28b mut* animals of 36 weeks of age were processed for immunofluorescence as described in the methods section. No major anatomical or histological differences were observed between *Ccdc28b mut* and *wt* animals and cilia were readily observed projecting into the lumen of tubules in both genotypes. We quantified the number of cilia per field (assessing areas of comparable cell density) and cilia length. Although we could not find statistically significant differences neither in cilia number or length, we did observe a trend towards a reduction in cilia density in *Ccdc28b mut* preparations, both in the kidney cortex and medulla, compared to *wt* mice (Fig 2C and 2D). We also analyzed cilia in the brain. We evaluated amygdala, hippocampus CA1, dentate gyrus (S3 Fig) and performed an in-depth quantification of both cilia density and length in the striatum (Fig 3A and 3B). Cilia density was comparable between *Ccdc28b mut* and *wt* controls (Fig 3B). Unexpectedly however, we observed a mild, albeit statistically significant, difference in cilia length whereby cilia in *Ccdc28b mut* animals were longer than *wt* controls: 10.16 ± 2.67 μm and 9.38 ± 2.40 μm respectively (*P* = 0.0022; Fig 3B). Depletion of BBS proteins has been shown to result in impaired cilia localization of different receptors (see for example [52]). We analyzed the localization of melanin-concentrating hormone receptor 1 (Mchr1) in the hippocampus CA1 region. Mchr1 was readily observed in cilia both in *wt* control and *Ccdc28b mut* animals (S4 Fig). Thus, our results show that depletion of CCDC28B does not result in a global cilia defect but can provoke cilia length changes at least in some tissues or cell types.

### Ccdc28b mut animals do not develop retinal degeneration or obesity

To continue the characterization of *Ccdc28b mut* mice we focused on assessing two phenotypes that have been shown to be highly penetrant in both BBS patients [1] and different BBS mouse models (for example see [46, 48, 53, 54]): retinal degeneration and obesity. In different models (*Bbs2*, *Bbs4* and *Bbs12*) retinal degeneration has been shown to be progressive, first evident as a thinning of the outer nuclear layer (photoreceptors) by as early as 6 weeks of age and characterized by complete loss of the outer segment in older animals (7 months old in *Bbs4* KO animals; [54]). In contrast, *Ccdc28b mut* retinas in both 12 weeks and 9 months old animals presented normal structure including the photoreceptor layer (Fig 3C; 9-month-old retinas are shown).

**Fig 4:**
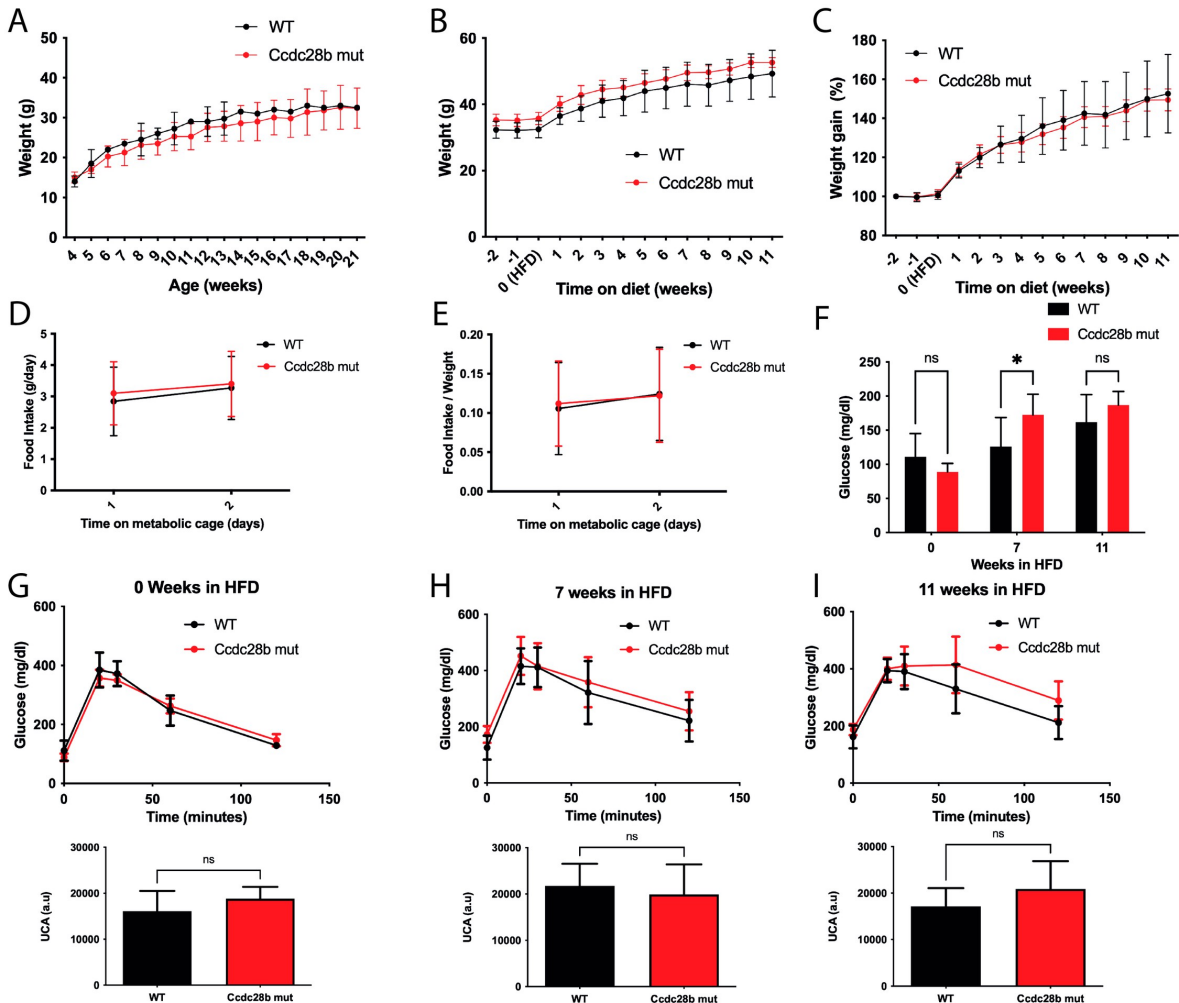
Metabolic characterization of *Ccdc28b mut* mice. **A)** Growth curve of *Ccdc28b mut* and *wt* control animals during a ND *ad libitum*. Both males and females were included in the analysis. **B)** Growth curve of *Ccdc28b mut* and *wt* controls during a HFD *ad libitum*. Both groups included seven males and at week -2 *wt* controls and *Ccdc28b muts* were on average 15 and 17 weeks old respectively. **C)** Weight gain curve of animals shown in B. Weight is normalized to the value at week -2. **D)** Food intake measured in metabolic cages. **E)** Food intake showed in D normalized to body weight. In all sections error bars correspond to mean and SD. **F)** Basal glycemia obtained from the corresponding GTT tests (panels G-I) measured at 8 weeks of age (normal diet), and after 7 and 11 weeks of HFD respectively. * = *P* < 0.05. **G-I)** GTT results for *Ccdc28b mut* and *wt* controls at 8 weeks of age (normal diet) (G), after 7 weeks of HFD (H) and after 11 weeks of HFD (I). Both glucose level curves and the quantification of the area under the curve are shown as mean ± SD of the analyzed animals.

*Bbs2*, *Bbs4*, *Bbs6*, and *Bbs12* KOs, as well as a *Bbs1*^*M390R/M390R*^ knock-in animals, develop obesity driven by hyperphagia [46–48,53–55]. To assess whether our *Ccdc28b mut* animals presented similar phenotypes we measured i) weight gain on normal diet, ii) weight gain on high fat diet (HFD), iii) food consumption and iv) systemic glucose handling (Fig 4). BBS mutant animals have been reported to be runted at birth and then rapidly start gaining weight at an increased rate. *Ccdc28b mut* animals presented a normal appearance at birth and gained weight at comparable rates to *wt* control animals when fed *ad libitum* on a normal diet. Mice were followed up to 21 weeks of age (Fig 4A). Next, we generated two groups of animals (n = 7 per genotype) for a HFD treatment. On average, *wt* controls and *Ccdc28b muts* were 15 and 17 weeks old respectively at the start of the experiment (week -2; Fig 4B), which likely explains a subtle (non-significant) initial weight difference (Fig 4B). Importantly, this difference remained constant along the duration of the HFD treatment (Fig 4B). In agreement with this, weight gain, analyzed as percentage of weight compared to the value at week -2 as 100%, was indistinguishable between the two genotypes (Fig 4C). Therefore, we did not observe significant weight differences between *Ccdc28b mut* and *wt* control animals either in normal diet or HFD (Fig 4A–4C). Accordingly, studying our mice in metabolic cages did not reveal any signs of increased food intake (Fig 4D and 4E). We also evaluated systemic glucose management by measuring glucose blood levels (basal glycemia) after 16 hours of starvation and performing glucose tolerance tests (GTT) before starting the HFD and at two time points during the treatment (7 and 11 weeks in HFD respectively). *Ccdc28b mut* mice showed significantly elevated basal glucose levels after 7 weeks on HFD (Fig 4F). By 11 weeks of HFD the difference was lost but mainly due to an increase in the glucose basal levels in *wt* control animals (Figs 4F and S5). In the GTTs we did not observe statistically significant differences although *Ccdc28b mut* mice showed a trend towards an impaired GTT response, particularly after 11 weeks on HFD (Fig 4G–4I). Overall, our results show that *Ccdc28b mut* animals do not present hyperphagia or obesity but show a mild phenotype related to systemic glucose management.

### Ccdc28b mut animals present autism-like behavioral phenotypes

Behavioral phenotypes have been reported in BBS patients [1,56]. In agreement with these documented observations, social dominance defects and anxiety related responses have been well documented in different BBS mouse models using standardized tests such as open field, light-dark box test and social dominance tube test [46–49]. Compulsive obsessive behavior and phenotypes similar to autistic spectrum disorder (ASD) have also been observed in BBS patients [1,56] but have been less studied in mouse models. Importantly, Kerr and colleagues assessed the behavioral phenotypes in a cohort of twenty-four confirmed BBS patients, demonstrating a high incidence of symptoms associated with autism [44]. In this context, we asked whether *Ccdc28b mut* mice displayed behaviors that could be relevant to BBS.

**Fig 5:**
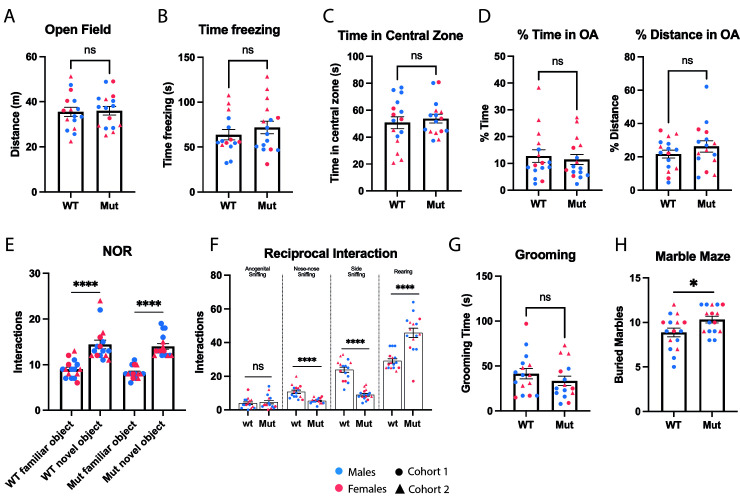
Evaluation of behavioral and social phenotypes in *Ccdc28b mut* mice. An open-field test was performed, and different parameters were scored: **(A)** total distance traveled, **(B)** time freezing and **(C)** time in the periphery *vs* center of the field. No differences were observed between *wt* and *Ccdc28b mut* mice. **D)** Results from elevated plus maze test showed no signs of anxiety as *wt* and *Ccdc28b mut* mice spent comparable amounts of time, and traveled comparable distances, in the open arms. **E)** The Novel Object Recognition test showed that there is no alteration in memory in *Ccdc28b mut* mice, as they presented an equally higher number of interactions with novel objects when compared to *wt* mice. **F)** Reciprocal Social Interaction Test resulted in significant differences in nose-nose, side sniffing, and rearing, consistent with an ASD-related phenotype, while no differences were found in anogenital sniffing. **G)** No differences were observed in grooming time between *Ccdc28b mut* and *wt* control animals. **H)** Marble burying test showing that *Ccdc28b mut* buried more marbles than *wt* animals, a behavior consistent with an obsessive-compulsive phenotype. In all graphs female and male mice are shown together: light blue indicates males and light red shows females. Triangles were used to indicate the five females analyzed in the second cohort (see Methods). All error bars correspond to mean and SEM. * = *P* < 0.05; **** = *P* < 0.0001.

We started our analysis of *Ccdc28b mut* animals performing an open field test to assess exploratory activity and overall movement. We analyzed both female and male animals and did not find significant differences in total distance traveled between *Ccdc28b mut* and *wt* mice (Fig 5A). We also evaluated time freezing and time in the periphery versus center of the field where we did not find significant differences between genotypes on either females or males (Fig 5B–5C). Next, we evaluated anxiety directly by performing the elevated plus maze test (EPM). *Ccdc28b mut* animals spent comparable amounts of time, and traveled comparable distances, in the open arms as *wt* control animals (Fig 5D). To directly test for alterations in hippocampal functions we performed a Novel Object Recognition (NOR) test. In this assay we evaluated memory by presenting individual animals with two objects for 10 min, and 24 hours later exchanging one known object for a novel one: the number of interactions of each mouse with each object (known *vs* novel) was quantified as described in methods. Both *Ccdc28b mut* and *wt* controls showed a significantly higher number of interactions with the novel object (Fig 5E).

Next, to study the impact of *Ccdc28b* in ASD behaviors often observed in mouse models of ASD [57], we performed a reciprocal social interaction test, a social dyadic test where the interrogated animal is presented with a previously unknown mouse to then quantify natural social behaviors. In this assay we evaluated anogenital, nose-nose and side sniffing, as well as grooming and rearing, measuring the number (frequency) of such behaviors in a period of 10 minutes. While *Ccdc28b mut* and *wt* controls did not differ in the frequency of anogenital sniffing or grooming, significant differences were readily observed for the other parameters: *Ccdc28b mut* animals presented a significant reduction in nose-nose and side sniffing and, in agreement with those results, also increased rearing (Fig 5F). Finally, we assessed stereotypical behaviors. Whereas *Ccdc28b mut* animals did not show differences in grooming when compared to *wt* controls (Fig 5G), *Ccdc28b mut* animals consistently buried more marbles than *wt* control animals in the marble burying test, a phenotype that was clearly observed both in males and females (Fig 5H). Altogether, our results show that while *Ccdc28b mut* mice neither present defects related to movement, grooming, nor signs of memory loss, they do show obsessive compulsive and mild social behavioral phenotypes.

## Discussion

*CCDC28B* was first identified as a second site modifier of BBS whereby a reduction in its levels, in conjunction with mutations in *bona fide* BBS genes, was shown to result in a more severe presentation of the syndrome in some families [37]. Also, we have shown previously that CCDC28B participates in the regulation of cilia in both cells and *in vivo* in zebrafish: depletion of *ccdc28b* resulted in cilia shortening and defective ciliogenesis in different zebrafish tissues and organs [40–42]. Consequently, knockdown of *ccdc28b* in the fish using primarily a morpholino approach resulted in several phenotypes that are characteristic of BBS and other ciliary mutants, such as a curved body axis, pigmentation defects, craniofacial malformations, and hydrocephaly ([40, 41] and references within). Therefore, while our previous results provided important functional information to understand CCDC28B biological role, they also led us to hypothesize that null mutations in *CCDC28B* could be sufficient to cause a ciliopathy such as BBS, or even the more severe Meckel-Gruber syndrome (MKS), two conditions with a high degree of genetic overlap [25, 40]. Importantly, the mutation described in patients, and shown to modify the presentation of the syndrome, is likely a hypomorphic mutation caused by a synonymous change affecting *CCDC28B* mRNA splicing thus resulting in reduced, but not abolished, CCDC28B levels [37].

We therefore decided to target *Ccdc28b* in the mouse and performed a characterization directing our attention to different phenotypes that have been reported previously for this gene (effect on cilia) and for BBS models. Overall, our data indicate that targeting *Ccdc28b* in the mouse is not sufficient to cause a strong ciliary defect and accordingly, does not cause phenotypes that are highly penetrant in different mouse BBS models, such as retinal degeneration or obesity. Thus, we did not obtain evidence to support a causal role for *CCDC28B* in BBS. We cannot rule out at this point the possibility of *Ccdc28b mut* animals presenting a predisposition to develop BBS like phenotypes. Older animals (we assessed mice up to 9 month of age) will have to be evaluated to test this possibility.

Our results however are fully compatible with a second site modifier role for *CCDC28B*. *Ccdc28b mut* mice did not show signs of retinopathy and did not develop obesity at least up to nine months of age. Moreover, using metabolic cages we were able to show that *Ccdc28b muts* are not hyperphagic. Interestingly however, when animals were fed on HFD, *Ccdc28b mut* mice show both a trend towards presenting a worst performance than *wt* controls in the GTTs and presented significantly higher glucose basal levels after 7 weeks on HFD. Thus, our results suggest a mild phenotype related to glucose handling. Interestingly, the consequences of BBS gene mutations on systemic glucose management are still not entirely clear. For example, while *Bbs4* KO animals have been shown to present impaired glucose handling, mainly due to defective insulin secretion [58], *Bbs12* KO animals have been shown to present an improved glucose metabolism [53]. Likewise, reports are showing that some BBS patients could present a dissociation between obesity and the development of type II diabetes [53,59]. Thus, it will be interesting to study whether variants in genes such as *CCDC28B* could contribute to modulating the presentation of obesity in BBS. Crossing this new *Ccdc28b mut* mouse line with available BBS mutants will allow us to start tackling this issue.

This work also underscores differences with our previous data working on cells and zebrafish where we observed clear ciliary phenotypes. Besides differences in models (culture cells *vs* zebrafish *vs* mouse), one possibility is that our *Ccdc28b mut* animals are not complete knockouts. We believe this to be unlikely considering our qRT-PCR, sequencing, and western blot results. Also, the mutant *Ccdc28b* mRNA could encode an 88 amino acid polypeptide which would only include the first 56 amino acids of CCDC28B. We favor a second scenario which relies on genetic compensation, a mechanism that has been shown to be particularly important to understand differences between targeting genes at the mRNA level versus at the genomic level [60,61]. This phenomenon has been well documented in zebrafish and other model organisms where the same gene has been targeted, for example, using morpholinos to block mRNA splicing/translation, and through genome editing, highlighting the complexity and plasticity of the genome. Oftentimes the phenotype of morphants is significantly more severe than that of genomic mutants and different mechanisms have been shown to explain these differences. For example, CRISPR-Cas9 mutant lines have been shown to present altered mRNA splicing thus bypassing the mutated residue/exon [62]. As mentioned, we could not find any evidence for alternative splicing in our mice. Importantly however, the compensation can also occur by rescuing the cellular function rather than a particular gene. For example, *egfl7* (endothelial extracellular matrix gene) genomic mutants, but not morphants, upregulate genes that are functionally related to *egfl7* [61]. Similarly, while targeting *CEP290* (*NPHP6*), a ciliopathy gene linked to BBS, in zebrafish at the mRNA level resulted in severe cilia-related phenotypes, only a mild defect restricted to photoreceptors was observed in genomic mutants. Interestingly, the authors found that the mild presentation of the later was associated with the upregulation of several genes associated to ciliary function [63]. Importantly, it has been shown that upregulation of compensatory genes is triggered by mutations that introduce a PTC in a mechanism that relies on NMD [64,65]. Complete deletion of *Ccdc28b* will be required to determine if this is the case in our model. However, rather than a weakness of this *Ccdc28b mut* mouse model, its mild phenotype could be seen as an opportunity to perform transcriptomic/proteomic studies attempting to dissect this compensation mechanism. Such data will likely shed important insight to understand the biological role of CCDC28B and the cellular/molecular pathways involved in cilia regulation.

Finally, we observed behavioral defects in our *Ccdc28b mut* animals and further work will be required to fully dissect this phenotype. For example, neonatal social interactions can have long lasting effects shaping the development of behavioral phenotypes (for some examples and a comprehensive review see [66–69]). In our experimental setup, *Ccdc28b mut* pups were raised by mutant mothers while *wt* controls were raised by *wt* mothers. Thus, the behavioral phenotype of *Ccdc28b mut* mice might be at least partially dependent on a maternal behavioral defect. Importantly, finding a maternal effect will not necessarily undermine the results presented here but rather expand them, given that the genotype will still likely contribute significantly to the phenotype in adult mice, and because it will indicate that *Ccdc28b mut* females present a social interaction maternal phenotype. Different breeding schemes as well as cross-fostering experiments [70] will be needed to complement the studies presented here and continue dissecting the role of *Ccdc28b* in behavior.

Anxiety, social dominance and associative learning defects, have been reported in different BBS mouse models [46–49]. For example, it was shown that BBS mice present problems in context fear conditioning due to impaired neurogenesis [49]. Our *Ccdc28b mut* animals did not show differences with *wt* controls in the open field, the EPM, or the NOR tests, thus ruling out significant locomotor, anxiety, and memory dysfunctions. *Ccdc28b mut* animals however did show social interaction defects as well as stereotypical phenotypes often observed in mouse ASD models. Interestingly, ASD-like behaviors have been described in BBS patients (see for example [43–45]). Moreover, although historically reported as a rare presentation, a comprehensive study of behavioral phenotypes in twenty-four BBS patients determined that autism related symptoms were present in 77% of cases [44]. Thus, it is tempting to speculate that decreased CCDC28B function could contribute to modulate the penetrance, the expressivity, or both, of ASD-like phenotypes in BBS patients, a possibility that will require further studies. In addition, one *de novo* mutation and several inherited missense variants in *CCDC28B* have been reported in the Simons Simplex autism cohort [71,72]. While it is difficult to dissect the individual contribution of CCDC28B towards autism features in these individuals, additional studies in larger cohorts, as well as functional evaluation of missense changes will be required to address the intriguing possibility of *CCDC28B* contributing to ASD behavioral features in humans.

Our *Ccdc28b mut* mouse model could provide an opportunity to study cellular/molecular aspects of ASD-like phenotypes. While our results in mice demonstrate a direct link between *Ccdc28b* and the development of behavioral phenotypes, whether these phenotypes are caused by a CCDC28B-dependent cilia defect and/or CCDC28B extra-ciliary functions is still not known. For example, it was shown recently that CCDC28B plays a role in immune synapse assembly by regulating T-cell antigen receptor, importantly, by affecting actin polymerization and endosomal trafficking, cellular processes that might prove relevant in the context of neuronal homeostasis [73]. In our analysis of the brain striatum, we observed that cilia were longer in mutants than controls. While this elongation was subtle, the difference was statistically significant. Two points regarding this cilia phenotype. First, all our previous data have clearly shown that CCDC28B plays a pro-ciliogenic role, whereby its depletion results in shortened cilia. Thus, one possibility is that the observation of longer cilia in striatum could be underscoring differences between cell types. It is tempting to speculate however that this slightly elongated cilia could be an indirect consequence of genetic compensation and the upregulation of pro-ciliogenic genes in the absence of CCDC28B. Further studies will be required to test this possibility and dissect whether depletion of CCDC28B may also affect the function of cilia directly. The second point relates to the potential physiological relevance of this finding. Wang and colleagues recently published a work using induced pluripotent stem cell-derived (iPSC) neurons obtained from both BBS patients and controls where they show that mutations in BBS genes affect neurite outgrowth and neuronal energy homeostasis. Interestingly, BBS mutant iPSC-derived neurons presented elongated cilia [74]. Observing elongated cilia in a region of the brain that has been linked to the development of ASD conditions, like the striatum [75, 76], suggests the possibility of cilia playing an active role in the development of these conditions. Further work will be needed to determine whether this is the case. The mouse line presented here, together with other already available models, will likely contribute to further our still incomplete understanding of cilia in neuronal homeostasis, brain development, the establishment of neuronal circuitry and its impact on behavior [77–79], thus providing important insight to understand complex behavioral phenotypes.

## Methods

### Ethics statement

All experimental protocols were opportunely approved by the Institutional Animal Ethics Committee (protocol number 007–18), in accordance with national law 18.611 and international animal care guidelines (Guide for the Care and Use of Laboratory Animal; [80]) regarding laboratory animal’s protocols.

### Animals

All animal procedures to generate the mutant line were performed at the SPF animal facility of the Laboratory Animal Biotechnology Unit of Institut Pasteur de Montevideo. Mice were housed on individually ventilated cages (Tecniplast, Milan, Italy) containing chip bedding (Toplit 6, SAFE, Augy, France), in a controlled environment at 20 ± 1°C with a relative humidity of 40–60%, in a 14/10 h light-dark cycle. Autoclaved food (Labdiet 5K67, PMI Nutrition, IN, US) and autoclaved filtered water were administered *ad libitum*. A C57BL/6J mouse colony was bred and maintained in our institutional facility and was used to both obtain zygotes for genome editing and to generate *wt* control animals. Both *wt* controls and *Ccdc28b mut* animals were housed in the same conditions and in the same room of the facility, thus minimizing environmental differences.

Cytoplasmic microinjection was performed in C57BL/6J zygotes using a mix of 30 ng/μl Cas9 mRNA (Synthego, Menlo Park, CA, US), and 15 ng/μl of each sgRNA (2 guides were used) (Synthego), diluted in standard microinjection buffer. Viable embryos were transferred into the oviduct of B6D2F1 0.5 days post coitum (dpc) pseudo-pregnant females (25 embryos/female in average), following surgical procedures established in our animal facility [81]. For surgery, recipient females were anesthetized with a mixture of ketamine (100 mg/kg, Pharmaservice, Ripoll Vet, Montevideo, Uruguay) and xylazine (10 mg/kg, Seton 2%; Calier, Montevideo, Uruguay). Tolfenamic acid was administered subcutaneously (1 mg/kg, Tolfedine, Vetoquinol, Madrid, Spain) to provide analgesia and anti-inflammatory effects [82]. Pregnancy diagnosis was determined by visual inspection by an experienced animal caretaker two weeks after embryo transfer, and litter size was recorded on day 7 after birth. Pups were tail-biopsied and genotyped 21 days after birth, and mutant animals were maintained as founders. *Ccdc28b mut* animals are fertile and thus the colony was maintained by crossing homozygous mutant animals.

### RNA Isolation and qRT-PCR

Tissues were homogenized in TRIzol (Invitrogen) for RNA extraction according to the manufacturer’s protocol. Reverse transcription was done using SuperScript II RT (Invitrogen) and qRT-PCR was performed using SYBR FAST Universal 2X qPCR Master Mix (Kapa) on a QuantStudio 3 RT-PCR System (Thermo Fisher Scientific). All samples were run in triplicate and the CT value was normalized to calculate relative expression of each gene. The fold expression was calculated using the ΔΔCt method with *Gapdh* as a reference gene.

### Western blotting

Tissues were lysed using RIPA buffer (25mM Tris pH 8.0, 150mM NaCl, 1% NP-40, 0.1% SDS, 1% sodium deoxycholate) supplemented with a protease inhibitor cocktail (Sigma). Protein concentrations were determined using the BCA Protein Assay Kit (Thermo Fisher Scientific) and 100μg of total protein were loaded into SDS-PAGE gels, transferred to PVDF membranes and probed with anti-CCDC28B (Invitrogen, 1/1000) overnight at 4°C. HRP-conjugated secondary antibody was used.

### Cell culture and immunofluorescence

Mouse embryonic fibroblasts (MEFs) were obtained from E13.5-E14.5 following standard procedures and maintained sub-confluent in DMEM Glutamax with 10% FBS, Hepes 10mM, penicillin 10000 U/mL and streptomycin 10000 μg/mL (Maintenance Medium, MM) under controlled conditions at 37° C with 5% CO_2_. For immunofluorescence studies cells were cultured on glass coverslips and at 80% confluency, MM was replaced with medium containing 0.5% FBS for 24 h to stimulate ciliation. Cells were fixed with 4% paraformaldehyde (PFA) in 0.1 M phosphate buffer saline (PBS), permeabilized with 0.1% Triton-X100, blocked with 5.5% FBS and stained with anti-gamma and anti-acetylated tubulin primary antibodies (Sigma) followed by the corresponding secondary antibodies conjugated to AF488 or TMRM (Invitrogen). Nuclei were stained with DAPI (Invitrogen). Images were taken in a Zeiss LSM 880 confocal microscopy. Eight randomly selected confocal fields from cultured MEFs from at least two *Ccdc28b mut* and two *wt* embryos were analyzed. Cilia length was measured using the freehand ROI selection tool of the FIJI image processing package [83]. The proportion of ciliated cells was calculated by counting the number of cells showing γ-tubulin and anti-acetylated tubulin staining over the total number of nuclei.

### Brain, kidney and eye histological and immunofluorescence analysis

The perfusion of the mice was performed during the light (resting) phase of the sleep-wake cycle (between 12:00 and 16:00 h, lights on at 7:00). The animals were anaesthetized with ketamine/xylacine (90 and 14 mg/kg, respectively) and perfused with PBS followed by 4% PFA. Brains, kidneys and eyes were immediately dissected out and fixed by immersion in 4% PFA overnight (ON). Thereafter, the brains were cryoprotected in 30% sucrose solution in 0.1 M PBS for 48 h and frozen. Coronal sections (30 μm) were obtained by a cryostat (Leica CM 1900, Leica Microsystems, Nussloch, Germany). Sections containing, hippocampus, amygdala and striatum (based on The mouse brain atlas; Paxinos & Franklin 2000) were collected and stored in an anti-freeze solution at −20°C until immunostaining procedures were performed. Kidneys and eyes were embedded in paraffin and cut into 6 micron slices. Eye sections were dewaxed, rehydrated and stained with Hematoxilin and Eosin following standard procedures.

Cilia were detected in brain and kidney sections by immunofluorescence. Renal cilia were stained as previously described [84], using boiling in 10 mM citrate buffer, pH6, for antigen retrieval and anti-acetylated tubulin (Sigma) 1/300 in PBS containing 0.5% of normal goat serum and 0.05% Tween20, for cilia detection. Goat anti-mouse Ig coupled to AF488 (Thermo) 1/1000 was used as secondary antibody and DAPI (Thermo) 1/5000 for nuclear staining. Cilia staining in brain sections was performed by detecting Type III adenylyl cyclase (AC-III). Briefly, free-floating sections were incubated with rabbit anti-AC-III primary antibodies 1/500 (Santa Cruz Biotechnologies) in PBS plus 0.3% Triton (PBS-T) and normal donkey serum (NDS) 1.5% for 48 h at 4°C. Then, the sections were incubated with biotinylated donkey anti-rabbit (DAR) 1/600 (Jackson ImmunoResearch) in PBS-T and NDS 3% for 90 min. Afterward, they were incubated with streptavidin-Alexa fluor 555 conjugate 1/2000 (Molecular Probes) in PBS for 2 h. Finally, the sections were mounted in Superfrost Plus slides with Vectashield (Vector Labs) and Hoechst was included to visualize nuclei. Negative controls consisted of omission of the primary antibodies. We obtained three 20x microphotographs of each region of interest. The level of ciliation was quantified assessing number of cilia per field comparing fields of similar cell density. Mchr1 localization was studied by immunofluorescence using a similar protocol and using a rabbit anti-Mchr1 monoclonal antibody (ThermoFisher Scientific). Images were obtained in a confocal microscopy Zeiss LSM 880.

### *In vivo* metabolic studies

The animals used in this study were raised and maintained according to standard protocols that were approved by the ethical committee at the Institut Pasteur de Montevideo (protocol number 003–19). Mice were fed *ad libitum*, first with a normal control diet (ND, Labdiet 5K67, PMI Nutrition, IN, US) and then with a high-fat diet (HFD, Test Diet 5TJN). Body weight was recorded weekly. For the weight gain control in ND a group of 4 *wt* (2 males and 2 females), and 7 *Ccdc28b mut* (4 males and 3 females) mice were followed up from week 4 to week 21. For weight gain in HFD a group of 7 *wt* and 7 *Ccdc28b mut* (all male) from 15–17 weeks of age were used to start the experiment (week -2), then changed to HFD (week 0) and maintained in that diet for 11 weeks when all mice were euthanized. For glucose tolerance testing, mice were starved for 16 h before receiving a single intraperitoneal glucose injection (1,5 g/kg). Glycemia was measured from tail vein blood using a hand-held glucometer (Accu-Chek, Roche). For food intake measurements, mice were transferred to metabolic cages, and after 24 h of adaptation, food was weighed every 24 h for three consecutive days.

### Behavioral analyses

Behavioral assays were conducted in a total of 32 mice, all between 11 and 13 weeks old, divided as follows: 8 *wt* males, 8 *Ccdc28b mut* males, 8 *wt* females and 8 *Ccdc28b mut* females. Cohort 1 consisted of all 16 males and 3 females of each group, while cohort 2 consisted of 5 females of each group. All assays were recorded using Any-maze software, except the marble maze test, and for all assays where automatization was not possible, videos were scored by investigators blinded to genotype.

### Open field

Locomotor activity was assayed in an open field opaque white plexiglass chamber (39 x60x50 cm), where animals were left for 6 min sessions to explore freely and afterwards were returned to their home cages. Any-maze software was used to measure total distance, time freezing and time in central area.

### Marble maze

Marble maze test can be used to assess repetitive, compulsive-like behaviors [85]. Briefly, each individual mouse was placed in an arena (15x27x20 cm) for 30 min, with a 5 cm layer of clean bedding, where 12 opaque glass marbles were distributed evenly. To be scored as buried, marbles had to be at least 50% covered by bedding.

### Grooming

Grooming can be used as a repetitive behavior assay [86]. Cages were left without bedding to eliminate digging, which can be a competing behavior [87]. Mice were placed individually in standard mouse cages (15x27x13 cm). Sessions lasted 20 min, with the first 10 min being unscored as a habituation period. During the second 10 min of the session, cumulative time spent grooming was scored manually by an investigator uninformed of genotype.

### Reciprocal interaction

The reciprocal interaction test was used to assess social behaviors and interactions towards an age- and sex matched partner. To this end, mice were placed in standard mouse cages (15x27x13 cm), and interactions were recorded for 10 min, which is the period during which most social interactions happen [87]. The test was recorded using any-maze software and scored manually. Parameters of social behaviors measured were anogenital sniffing, nose-nose sniffing, side sniffing, self-grooming, and rearing [88,89].

### Elevated plus maze

The Elevated Plus-Maze test is used as a model of anxiety [90,91]. The apparatus consists of four arms (29x7 cm), two of them with walls 16 cm high (closed arms), and the other two have no walls, the open arms, the center that connects them is 8x8 cm. This maze was placed 50 cm from the floor, and mice were placed in the middle, facing an open arm and left to explore freely for 5 min. Time and distance spent in open arms was scored using Any-maze software.

### Novel object recognition

This test is constructed to assess the mouse’s ability to recognize a novel object in the environment, and is divided in three phases: habituation, familiarization, and test phase [92]. Briefly, on the first day, for the habituation period, animals were placed in an empty arena (25x25x35 cm) and left to explore for 5 min before returning to their home cages. 24 h later, the familiarization phase was performed, in which two identical objects were placed in the arena, and each mouse was allowed to explore for 10 min. Finally, on the third day, the test phase consisted of the mouse in the same arena for 10 min, where one of the objects was switched for a novel object, previously unseen to the animal. During the last two phases, objects were placed in opposite and symmetrical corners of the arena [92, 93]. Behavior was scored for the first 2 min of the test phase, or until the mouse had interacted with both objects a total of 20 times.

### Statistical analyses

All data were analyzed using GraphPad Prism 9. Unpaired t-test was used to compare the fold expression in the qRT-PCR analysis. For GTT analysis, the area under the curve was calculated in GraphPad Prism 9, and then compared between both groups using the unpaired t-test. Basal glucose between both groups at different times was compared with the 2-way ANOVA with multiple comparisons. The proportion of cilia positive cells was calculated by counting the number of cilia with clear acetylated-tubulin signal over the total number of nuclei and the comparison between the different samples was performed using a test of Hypothesis specific for comparison of two proportions (hypothesis test for proportions). For the analysis of cilia length, and number of cilia per field, we first tested the data sets for normal distribution, using the Shapiro-Wilk test. If normal distribution was proved, unpaired t-test was used for comparison, and if data did not have a normal distribution, comparisons were performed using the Mann-Whitney test. In all behavioral analysis (OF, MM, GR, RI and NOR) we first tested the datasets for normal distribution, using the Shapiro-Wilk test and identified outliers. After normal distribution was probed and outliers excluded, unpaired t-test was used to compare the two groups. In all cases, differences were considered significant when *P* values were smaller than 0.05.

## Supporting information

S1 Fig Nanopore sequence alignments to the genomic *Ccdc28b* region. (PDF)

S2 Fig Full length western blot gels assessing the levels of CCDC28B in brain and muscle. (PDF)

S3 Fig Brain cilia analysis. Confocal images showing A) amygdala (bar = 50 μm), B) hippocampal CA1 (bar = 50 μm), C) dentate gyrus (bar = 50 μm). DAPI (magenta) and anti-ACIII antibody (green) were used for nucleus and cilia visualization respectively.
(PDF)

S4 Fig MCHR1 localization in the pyramidal layer of CA1 region of the hippocampus. Three animals per genotype and two photos per animal were analyzed. The number of cilia per field was quantified.
(PDF)

S5 Fig Comparison of basal glycemia at three timepoints during the HFD experiment for each genotype (*Ccdc28b mut* and *wt*). (PDF)
