# Supplementary figures and images for: Generation and characterization of Ccdc28b mutant mice links the Bardet-Biedl associated gene with mild social behavioral phenotypes
Source: PLoS Genet. 2022 Jun 2;18(6):e1009896. doi: 10.1371/journal.pgen.1009896 (PMC9197067; doi:10.1371/journal.pgen.1009896)

A

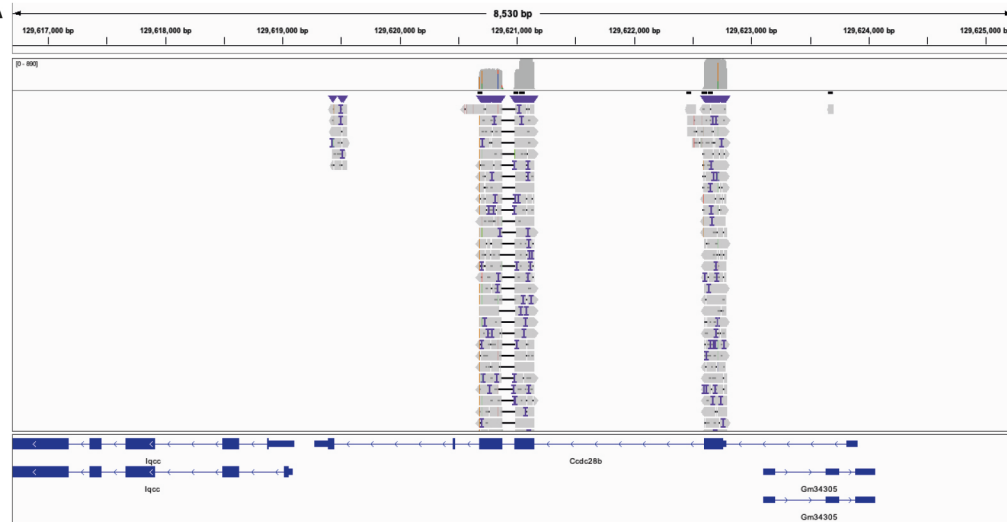

Liver WT

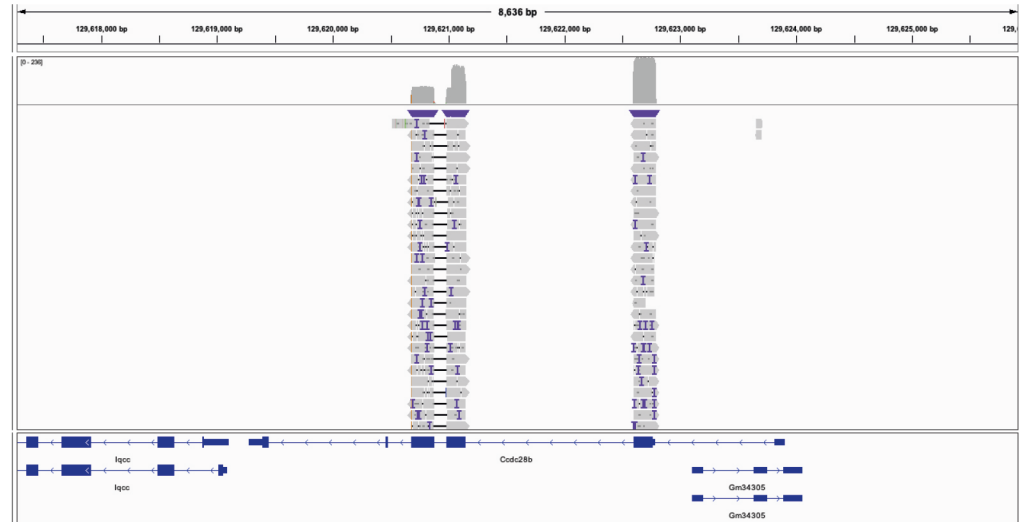

Liver Ccdc28b mut

B

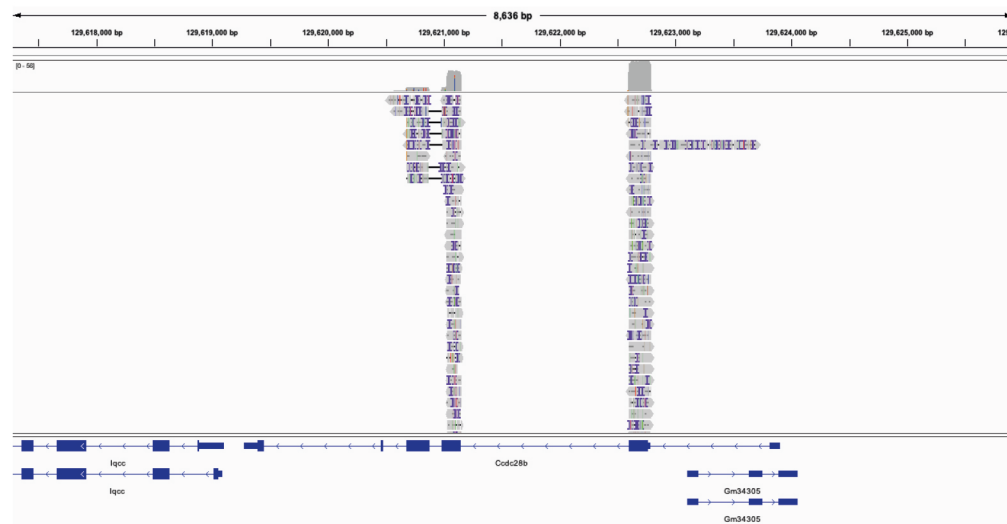

Muscle WT

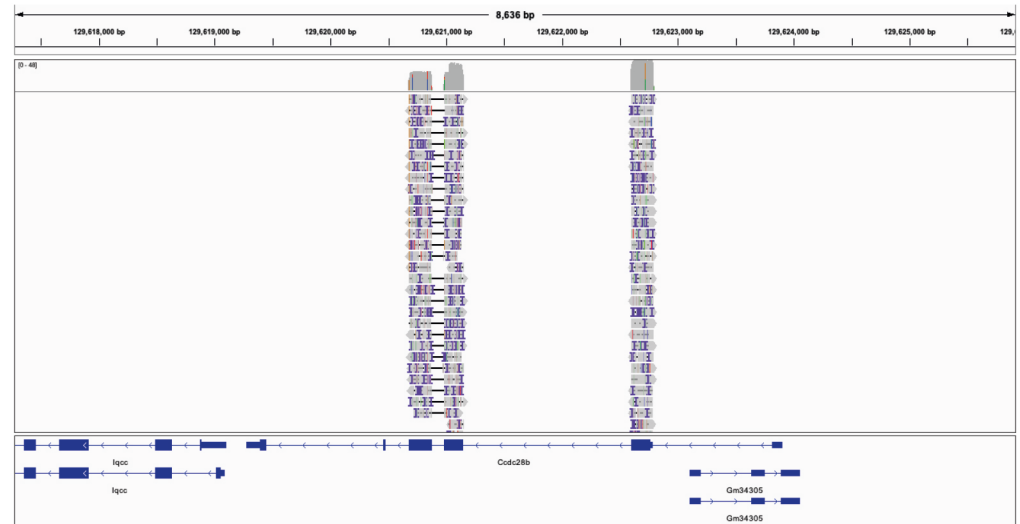

Muscle Ccdc28b mut

Supplement: S1 Fig — (PDF) [file pgen.1009896.s001.pdf]

A

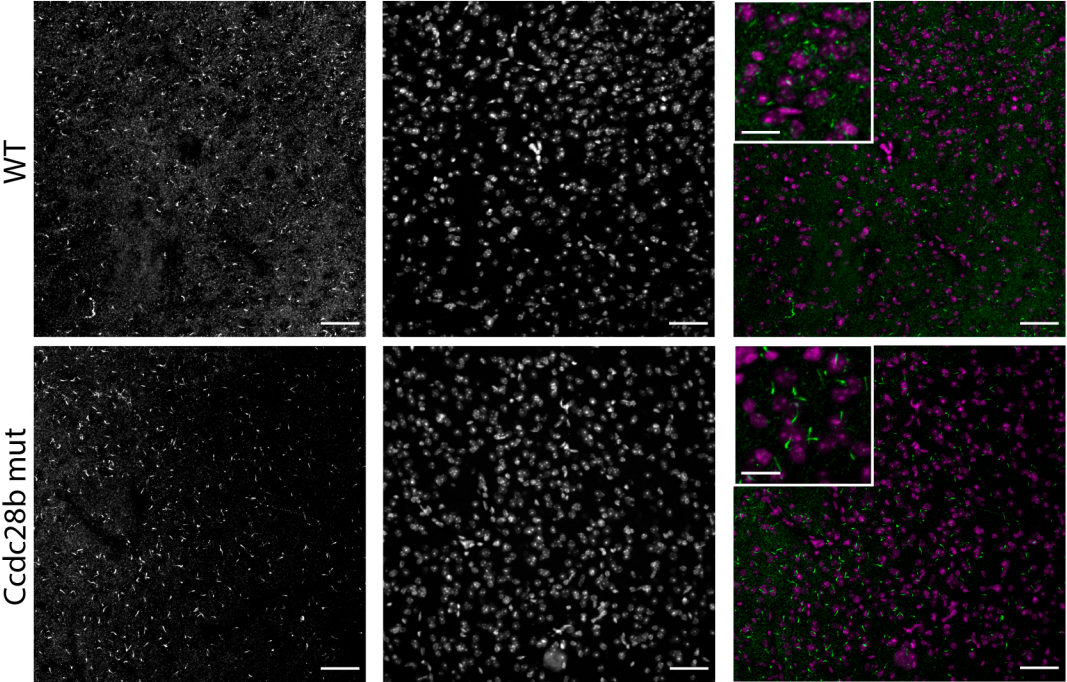

B

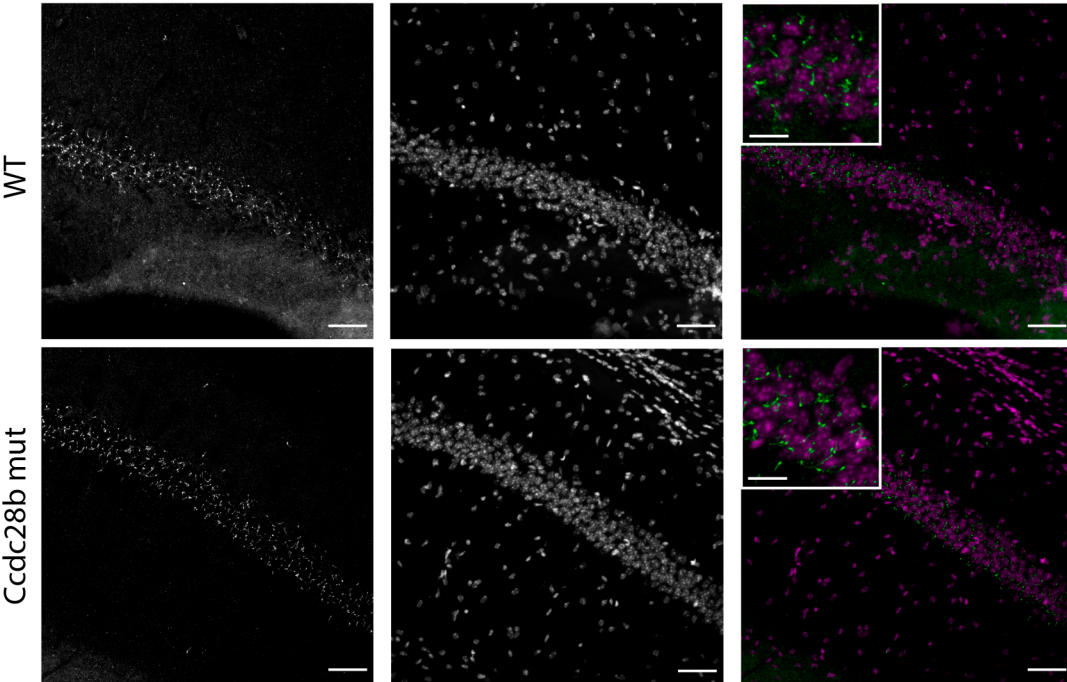

C

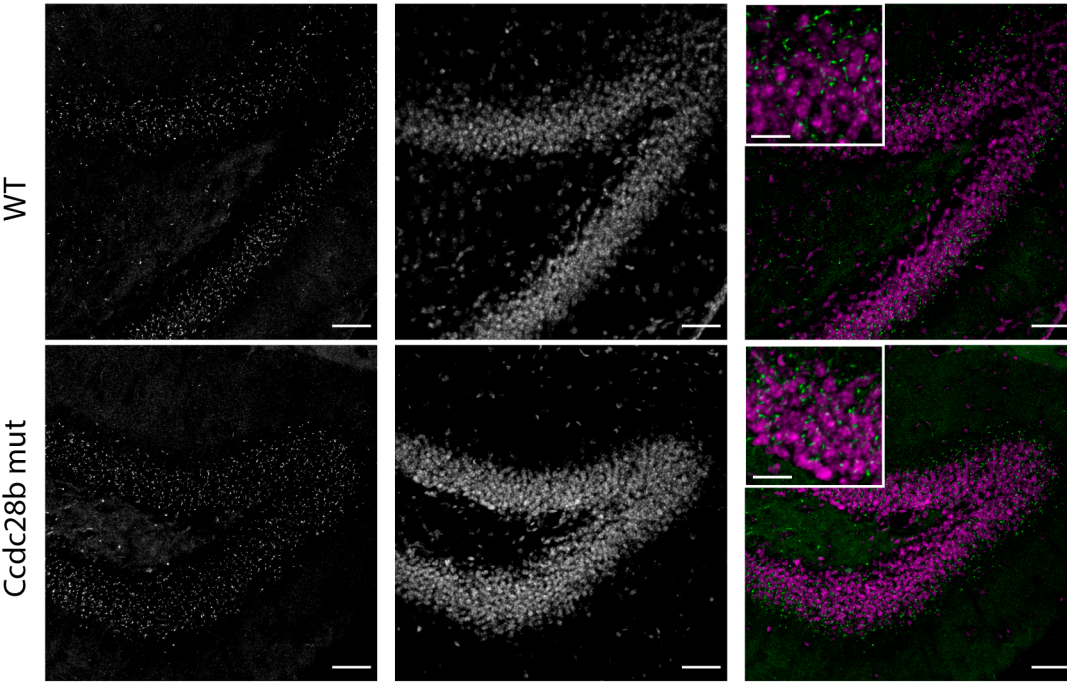

Supplement: S3 Fig — Confocal images showing A) amygdala (bar = 50 μm), B) hippocampal CA1 (bar = 50 μm), C) dentate gyrus (bar = 50 μm). DAPI (magenta) and anti-ACIII antibody (green) were used for nucleus and cilia visualization respectively. (PDF) [file pgen.1009896.s003.pdf]

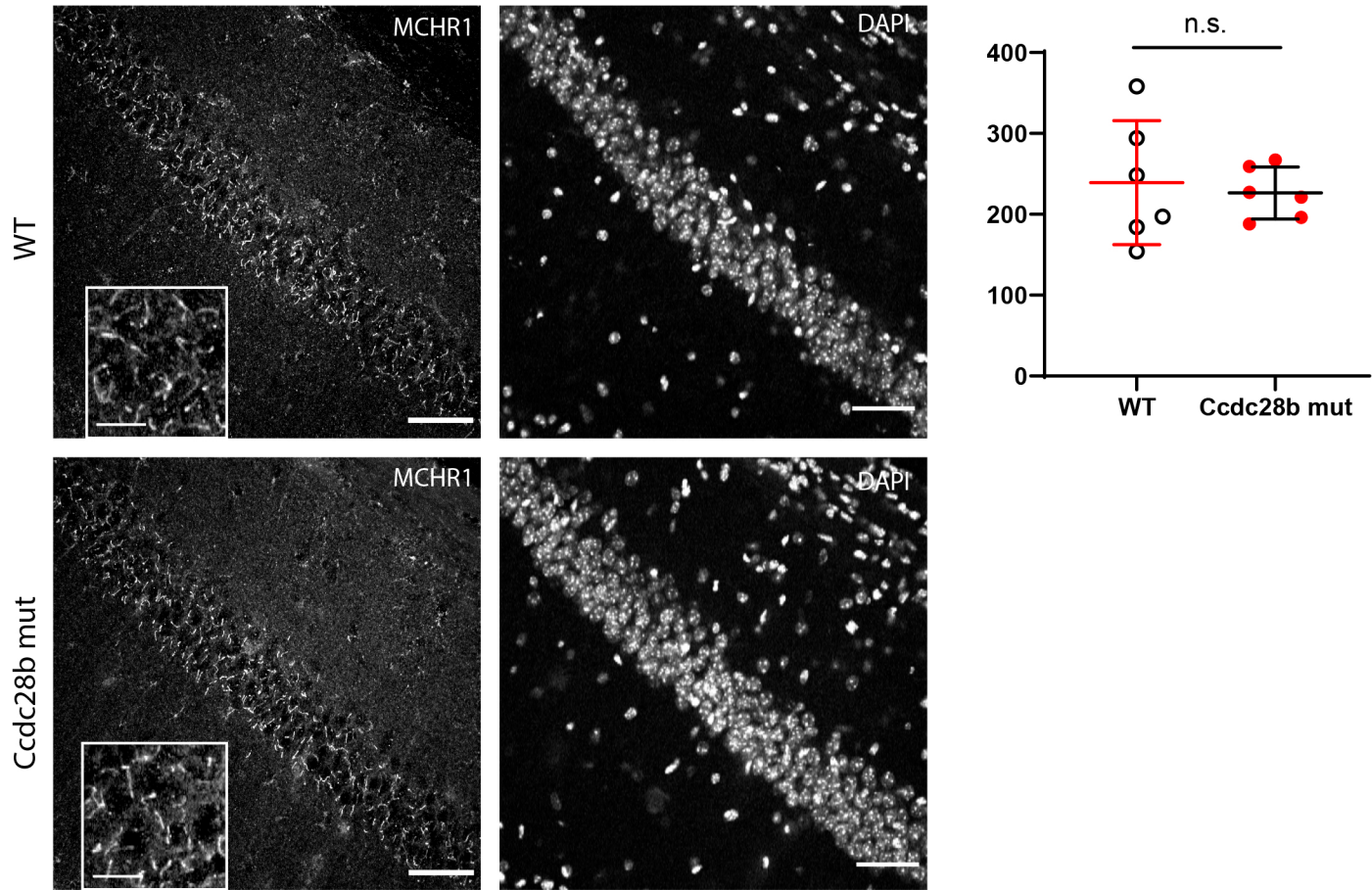

S4 Fig

Supplement: S4 Fig — Three animals per genotype and two photos per animal were analyzed. The number of cilia per field was quantified. (PDF) [file pgen.1009896.s004.pdf]

### WT Basal glucose

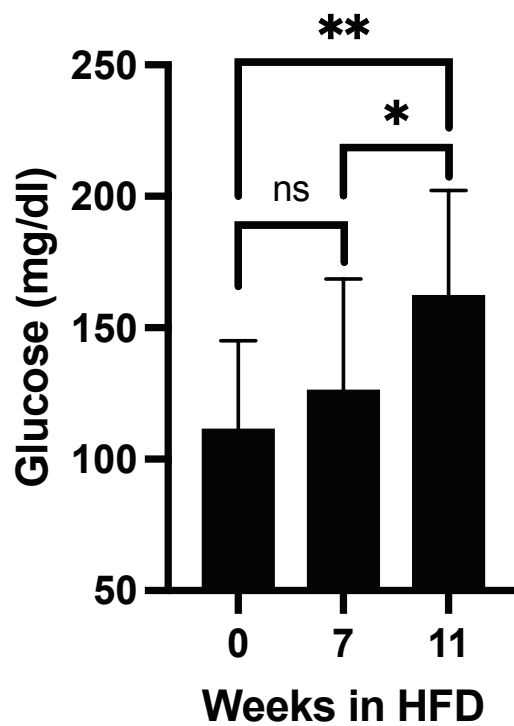

### Ccdc28b mut Basal glucose

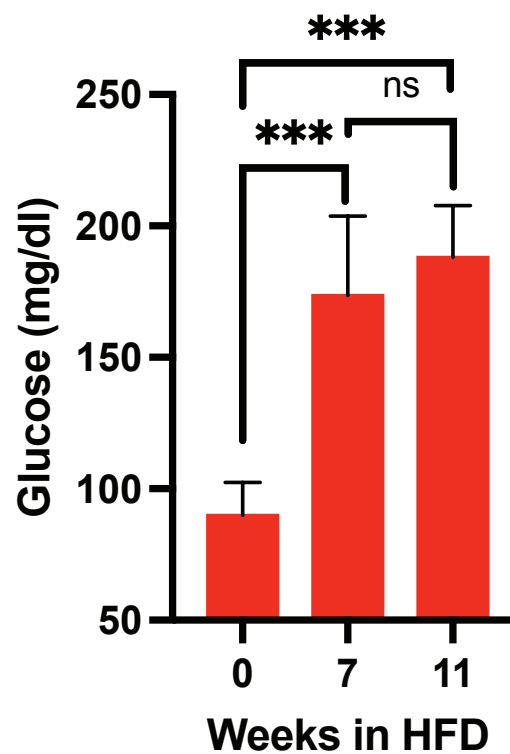

S5 Fig

Supplement: S5 Fig — (PDF) [file pgen.1009896.s005.pdf]
