# Supplementary material for: Generation and characterization of Ccdc28b mutant mice links the Bardet-Biedl associated gene with mild social behavioral phenotypes
Source: PLoS Genet. 2022 Jun 2;18(6):e1009896. doi: 10.1371/journal.pgen.1009896 (PMC9197067; doi:10.1371/journal.pgen.1009896)
Supplement: S2 Fig — (PDF) [file pgen.1009896.s002.pdf]

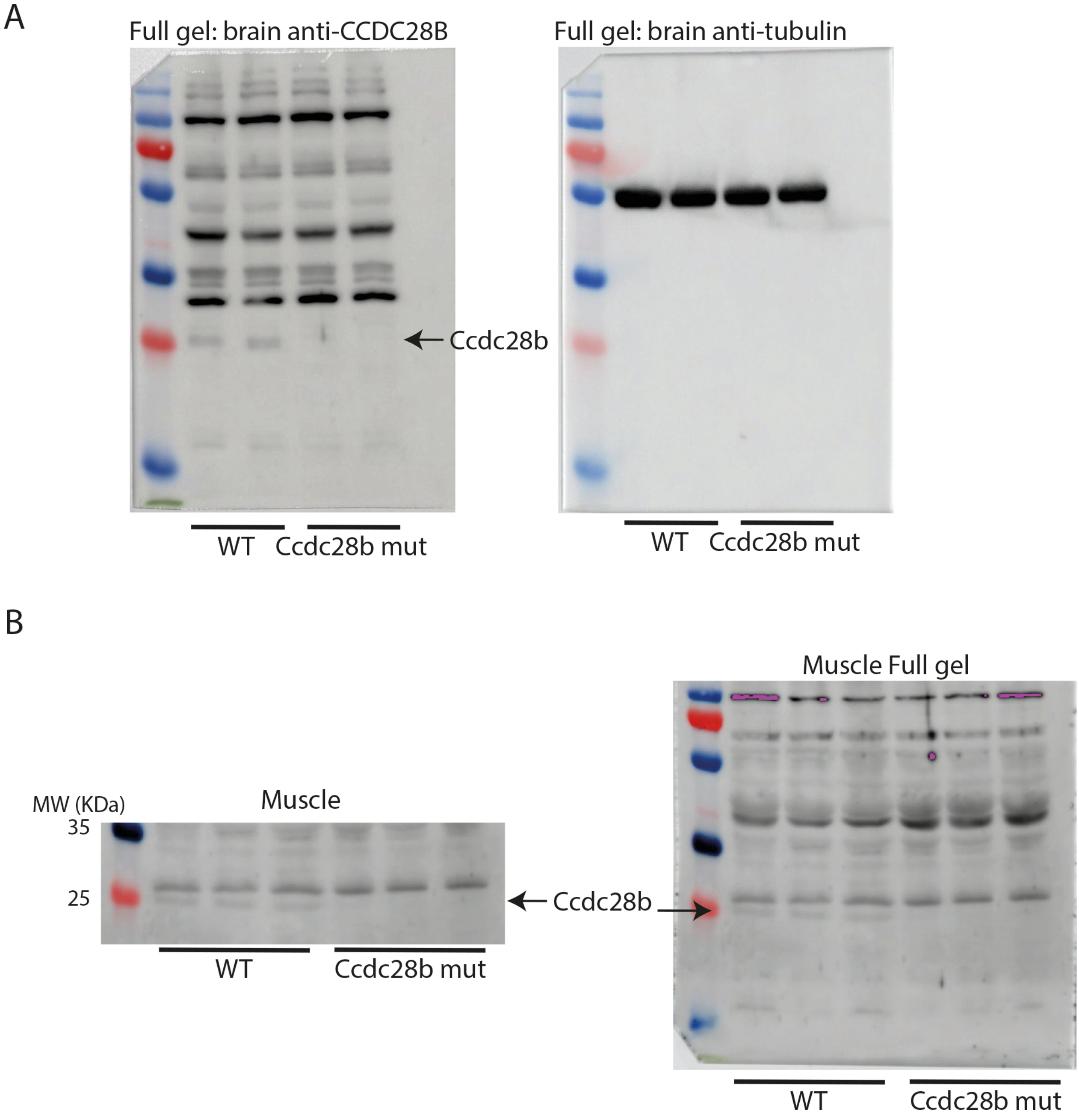
A
Full gel: brain anti-CCDC28B
Ccdc28b
WT
Ccdc28b mut
Full gel: brain anti-tubulin
WT
Ccdc28b mut
B
MW (KDa)
35
25
Muscle
Ccdc28b
WT
Ccdc28b mut
Muscle Full gel
WT
Ccdc28b mut

S2 Fig
